# Who do we think we are: interdisciplinary dissonance and the development of professional identity

**DOI:** 10.1186/1757-1146-3-S1-O3

**Published:** 2010-12-20

**Authors:** Judith Barbaro-Brown

**Affiliations:** 1 Durham University, Durham, UK

## 

The development of professional identity is something which all students entering a vocational profession will encounter, and this is particularly so within health care where health professionals are expected to work as part of a team rather than as individual and isolated practitioners. Alongside this, students will develop perceptions relating to the roles, responsibilities, and even behaviours of other team members, influenced by personal experience, social background, and the media. This is referred to as ‘construction of the other’ (Lingard et al, 2002), and it appears that once these constructs or impressions are formed, they are difficult to change. In some health provision situations there appears to be a divergence in these perceptions between team members which can lead to inter-disciplinary conflict, thereby potentially impacting on team performance. If a better understanding could be gained of how professional identity begins to develop in the early years of health-care/medical education, particularly in relation to the ‘self’ and the ‘other’, then perhaps educational strategy could be adapted and enhanced to help students and newly-qualified practitioners overcome potential difficulties when faced with working within the multi-disciplinary team. This paper aims to examine the influences which encourage the development of professional identity, and to discuss how educational strategy can be used to overcome this.
